# The Role of Forkhead Box Q1 Transcription Factor in Ovarian Epithelial Carcinomas

**DOI:** 10.3390/ijms131113881

**Published:** 2012-10-26

**Authors:** Min Gao, Ie-Ming Shih, Tian-Li Wang

**Affiliations:** 1 Department of Obstetrics and Gynecology, Qilu Hospital, Shandong University, 107 W. Wenhua Road, Jinan 250012, China; E-Mail: min.gao0620@gmail.com; 2 Departments of Pathology, Gynecology/Obstetrics, and Oncology, Program in Pathobiology, Johns Hopkins Medical Institutions, Baltimore, MD 21231, USA; E-Mail: ishih@jhmi.edu

**Keywords:** ovarian cancer, FOXQ1, survival, migration, invasion

## Abstract

The role of the forkhead box Q1 (FOXQ1) transcription factor in cancer pathogenesis has recently emerged. Overexpression of FOXQ1 has been found in a variety of human cancers, and its upregulation has been associated with poor prognosis in colorectal, breast, and non-small cell lung carcinomas. However, the molecular mechanism underlying how FOXQ1 contributes to ovarian epithelial carcinomas remains unclear. To this end, we analyzed gene expression levels in ovarian cancer tissues and cell lines and demonstrated a higher expression level of FOXQ1 in epithelial ovarian cancer cells than that in normal epithelial cells. We then used a human ovarian cancer cell line, SKOV3, which expressed a higher level of FOXQ1, as a cell model to investigate the biological effects of FOXQ1 by using RNA interference. Silencing of FOXQ1 expression using a shRNA knockdown approach affected the expression of several cell cycle regulators, leading to suppressed cell proliferation, reduced cell motility/invasion, and upregulation of epithelial cell markers and the downregulation of mesenchymal cell markers. Taken together, these results suggest that FOXQ1 expression is essential to maintain cell proliferation, motility/invasion, and epithelial-mesenchymal transition phenotypes in ovarian cancer cells.

## 1. Introduction

Ovarian epithelial cancers can be broadly grouped into two major types. Type I tumors are classified as low-grade serous carcinomas, clear cell carcinomas, endometrioid carcinomas, and mucinous carcinomas, whereas type II tumors mainly consist of high-grade serous carcinomas. Compared to type I tumors, type II carcinomas are more common and aggressive, and they usually present at advanced stages when the standard therapy is usually not effective [1]. In fact, high-grade serous carcinoma is the most common and lethal type of ovarian cancers. Recent studies have shown that high-grade serous carcinoma may arise from the fallopian tube epithelium rather than ovary, which may shed light on the early detection and prevention of ovarian cancers [2]. At the molecular level, type II tumors can be distinguished from type I neoplasms by the presence of frequent mutations in *TP53* gene and by the DNA copy number alterations including amplification of the cyclin E1 (*CCNE1*), *MYC*, and *NOTCH3* loci. High-grade serous carcinoma is also characterized by unique gene expression patterns; however, it remains largely unclear as to how individual genes and the pathways they controlled promote tumor progression.

While studying the transcriptome regulated by *NACC1*, a gene that encodes the NAC1 protein and participates in the pathogenesis of ovarian cancer recurrence and chemoresistance [3], we found that FOXQ1 was significantly upregulated by NAC1 in ovarian high-grade serous carcinomas. Ectopic expression of FOXQ1 reversed the NAC1 knockdown-mediated decrease in cell invasion (Gao *et al.*, manuscript in preparation). Therefore, the above results suggest a previously unreported role of FOXQ1 in ovarian cancer. FOXQ1 belongs to the Forkhead box (FOX) transcription factor superfamily, which is characterized by a conserved stretch of 110 amino acids that is responsible for DNA binding. There are a total of 17 FOX subfamilies (FOXA–FOXR) containing at least 41 unique proteins. Deregulation of FOX proteins such as FOXO, FOXM, FOXP, FOXC, FOXA, and FOXQ has been implicated in tumorigenesis, given that they are involved in a wide spectrum of biological activities including metabolism, development, differentiation, proliferation, apoptosis, migration, and invasion [4]. Specifically, the FOXQ1 gene, which encodes a protein of 403 amino acids, was first identified in 2001 [5], and its biological importance was highlighted by its involvement in embryonic stem cell biology and vertebrate development by acting as a downstream mediator of Hoxa1 and Hoxc13 [6,7]. Moreover, FOXQ1 is also known as a critical transcription factor that regulates epithelial-mesenchymal transition (EMT) [8,9], a developmental process that is often activated during cancer invasion and metastasis [10]. Given the potential role of FOXQ1 in cancer biology, the present study was undertaken to characterize its functional role in ovarian carcinomas.

## 2. Results and Discussion

### 2.1. FOXQ1 is Upregulated in Ovarian Cancer Tissues and Cell Lines

**Figure 1:**
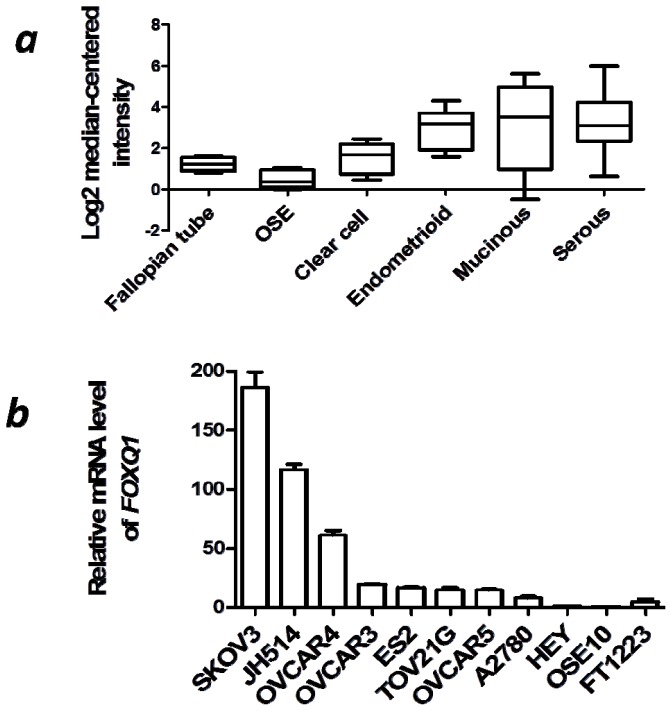
Expression of forkhead box Q1 (FOXQ1) in ovarian cancer tissues and cell lines. (**a**) *In silico* analysis indicated higher FOXQ1 expression levels in the epithelial ovarian cancers than those in normal epithelial tissues. The microarray data were normalized by log2 median-centered method in each category. (**b**) Quantitative RT-PCR analysis of *FOXQ1* mRNA levels in nine ovarian cancer cell lines (as indicated in the graph), one ovarian surface epithelial cell line (OSE10), and one fallopian tube epithelial cell line (FT1223). SKOV3 cells demonstrated the highest mRNA expression level of *FOXQ1*. The data were represented as fold-increase in *FOXQ1* expression of each cell line in comparison with the *FOXQ1* expression in OSE10 cells.

To evaluate the expression pattern of FOXQ1 in ovarian carcinomas, we selected the gene expression microarray dataset assembled by Lu *et al.*[11], because this dataset contains all of the major subtypes of epithelial ovarian cancers, as well as five pools of normal ovarian epithelial brushings and four normal fallopian tube mucosal scrapings—the two likely normal counterparts of ovarian cancer. We found that FOXQ1 was significantly overexpressed in both type I and type II ovarian cancers. The ovarian cancer subtypes including clear cell carcinoma, endometrioid carcinoma, mucinous carcinoma, and high-grade serous carcinoma showed a higher expression level of FOXQ1 (*p* < 0.01, *p* < 0.0001, *p* < 0.01, and *p* < 0.0001, respectively) compared with normal ovarian surface epithelium. When compared to normal fallopian tube tissue, FOXQ1 was overexpressed in endometrioid and high-grade serous types of ovarian carcinomas (*p* < 0.001 and *p* < 0.0001, respectively) (Figure 1a).

To further verify the above array-based results obtained from human ovarian cancer tissues, we compared the relative transcript levels of *FOXQ1* in a panel of nine ovarian cancer cell lines. An ovarian surface epithelial cell line (OSE10) and a fallopian tube epithelial cell line (FT1223) were used as normal controls. Real-time reverse transcription-polymerase chain reaction (qRT-PCR) analysis revealed that the expression of *FOXQ1* was upregulated in all ovarian cancer cell lines when compared with OSE10 cells (*p* < 0.01) and most of the cancer cell lines except HEY and A2780 when compared with FT1223 cells (*p* < 0.01) (Figure 1b). The SKOV3 cell line showed the highest expression level of *FOXQ1* among all ovarian cancer cell lines analyzed, and was therefore selected as an experimental cell model for assessing the function of FOXQ1 in ovarian cancer cells. Collectively, these data suggested that FOXQ1 expression was consistently higher in ovarian cancer cells than that in the normal epithelial cells, which implicated it may execute an important role in ovarian cancer development.

### 2.2. FOXQ1 Knockdown Reduces Cell Growth and Clonogenicity

**Figure 2:**
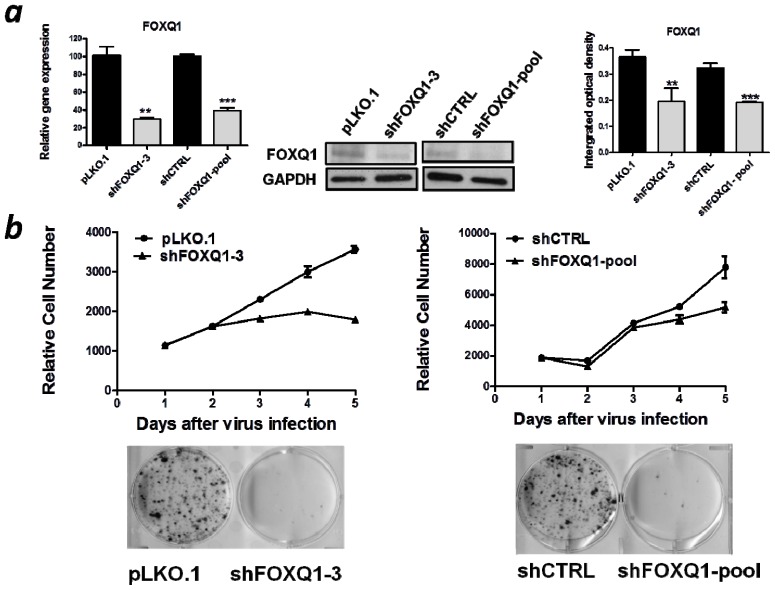
Growth inhibition of SKOV3 cells after FOXQ1 knockdown. (**a**) qRT-PCR (left) and western blots (middle) were performed to verify the knockdown efficiencies of the two FOXQ1 shRNA lentiviruses in SKOV3 cells. Relative mRNA levels were determined using glyceraldehyde-3-phosphate dehydrogenase (*GAPDH*) for data normalization. GAPDH protein expression was used as a loading control in the western blot analysis. The bar graphs (right) indicate the relative density of the FOXQ1/GAPDH western blot band signals measured by using the Image J software. (**p < 0.01, ***p < 0.001). (**b**) Growth curve analysis (upper panel) showed that inhibition of FOXQ1 using both shRNA approaches significantly decreased cellular proliferation compared with that of the control groups. Anchorage-dependent colony formation assay (lower panel) showed marked reduction in the number of colonies in the FOXQ1 knockdown SKOV3 cells.

To avoid the possible off-target effect associated with shRNA knockdown, we chose two different shRNAs: shFOXQ1-3 and shFOXQ1-pool. The knockdown efficiencies of both shRNAs were confirmed at the mRNA and protein levels in SKOV3 cells (*p* < 0.01) (Figure 2a). Next, we examined the effect of FOXQ1 knockdown on cellular growth. FOXQ1 or control shRNA-treated cells were subjected to a SYBR green-based cell proliferation assay. In addition, cells were also plated at a very low density to determine their clonogenicity. Our results demonstrated that suppression of FOXQ1 using either individual or pooled shRNA significantly decreased proliferation of SKOV3 cells and markedly inhibited colony formation compared with the corresponding control shRNA-treated SKOV3 cells (Figure 2b).

### 2.3. FOXQ1 Depletion Leads to G1/S Cell Cycle Arrest and Alters Cell Cycle Regulators

**Figure 3:**
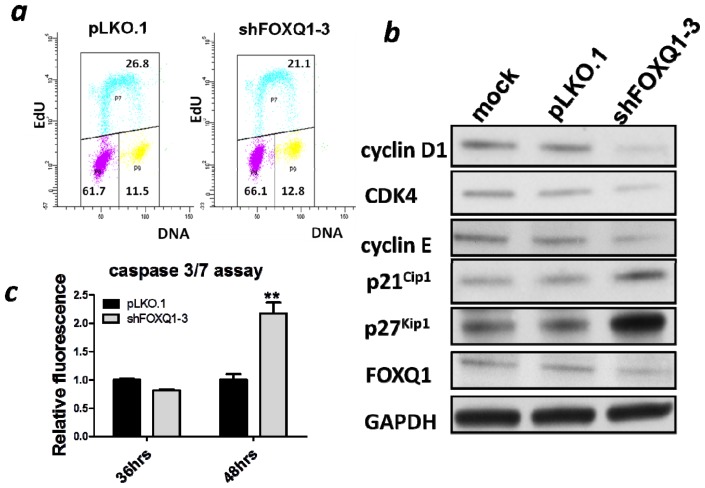
Effect of FOXQ1 knockdown on the cell cycle. (**a**) Cell cycle analysis showed downregulation of FOXQ1 expression in SKOV3 cells leaded to a decreased population of cells in the S-phase, whereas an increased population of cells in the G0/G1 phase. (P8, P7, and P9 represent G0/G1, S, and G2/M phases, respectively). (**b**) A panel of cell cycle-related proteins were analyzed using western blots. cyclin D1, CDK4, and cyclin E were downregulated after FOXQ1 knockdown, whereas p21^Cip1^ and p27^Kip1^ were upregulated compared with the control groups. (**c**) Apoptosis was detected by measuring the activities of caspase 3/7 using a commercial kit at both 36 h and 48 h after infection of SKOV3 cells with shFOXQ1-3 or control shRNA lentivirus. **p < 0.01.

To further investigate how FOXQ1 depletion suppressed cellular proliferation, we evaluated alterations in the cell cycle distribution of SKOV3 cells. Thirty-six hours after FOXQ1 knockdown, we began to observe an increase of cells in G0/G1 phase in FOXQ1 shRNA treated group compared to the control group (data not shown). While at forty-eight hours, we observed a modest but significant increase of cell population in the G0/G1 phase (61.7% in the control group *vs.* 66.1% in the knockdown group, *p* < 0.01) and a concomitant decrease of cell population in the S phase (26.8% in the control group *vs.* 21.1% in the knockdown group, *p* < 0.05) (Figure 3a). These results were compatible with a G0/G1 cell cycle arrest. To examine the effect of FOXQ1 knockdown on cell cycle regulators, we performed western blot analysis to determine the expression levels of several cell cycle regulators including cyclin D1, cyclin E, CDK4, p27^Kip1^, and p21^Cip1^. Interestingly, the levels of cyclin D1, cyclin E, and CDK4 were decreased after FOXQ1 depletion, whereas the levels of the cyclin-dependent kinase inhibitors (CDKIs) p27^Kip1^ and p21^Cip1^ were increased (Figure 3b). These data suggested that FOXQ1 expression controls the levels of cell cycle regulators, and in the absence of FOXQ1, these regulators can act in a concerted manner to prevent cell cycle progression.

In the present study, we also measured the activities of caspases 3 and 7 in order to examine whether FOXQ1 knockdown affected apoptosis. No significant effect on caspase activity was observed at 36 h after FOXQ1 suppression; however, the caspase activity started to increase at 48 h after FOXQ1 knockdown (Figure 3c). Because a G0/G1 block was detected at an earlier time point than apoptosis, induction of apoptosis was probably secondary to the G0/G1 block.

Aberration in the signaling of cell proliferation pathway is one of the most important features charactering all human malignancies [12]. In terms of how FOXQ1 regulates cell cycle, we showed that expressions of several cell cycle regulators were altered by FOXQ1 knockdown. Specifically, the positive regulators of the cell cycle, such as cyclin D1, CDK4, and cyclin E, were downregulated, whereas the negative regulators of the cell cycle, such as the CDKIs p21^Cip1^ and p27^Kip1^, were upregulated by FOXQ1 knockdown. In future studies, it would be interesting to determine whether FOXQ1, acting as a transcriptional regulator, controls promoter activities of those genes involved in cell cycle regulation. Nevertheless, FOXQ1 appears to modulate cell cycle progression through multiple effectors at different phases of the cell cycle. It should be pointed out that the role of FOXQ1 in cell cycle regulation is perhaps context-dependent, given that conflicting data have been reported in experiments performed on different cell types. For example, FOXQ1 knockdown was found to suppress, enhance, or impose no effect on proliferation of epithelial cell lines obtained from mouse mammary epithelium [13], human non-small cell lung carcinoma [14] and human mammary epithelium [9] respectively. From this perspective, it would be necessary that future efforts be focused on determining the effects of FOXQ1 knockdown in cell lines from different types of ovarian cancers.

### 2.4. FOXQ1 Knockdown Decreases Ovarian Cancer Cell Movement and Affects the Expression of Epithelial-Mesenchymal Transition (EMT) Markers

**Figure 4:**
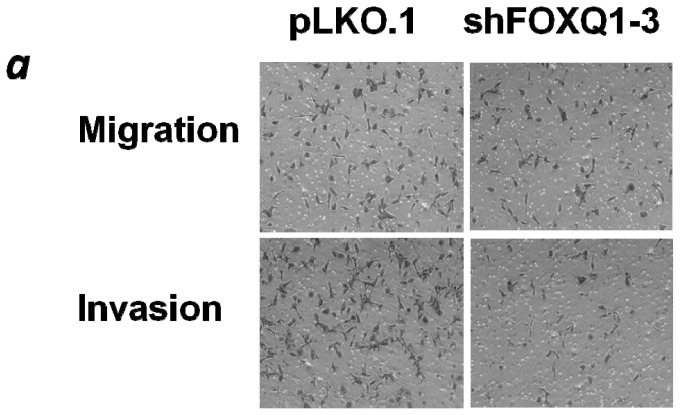

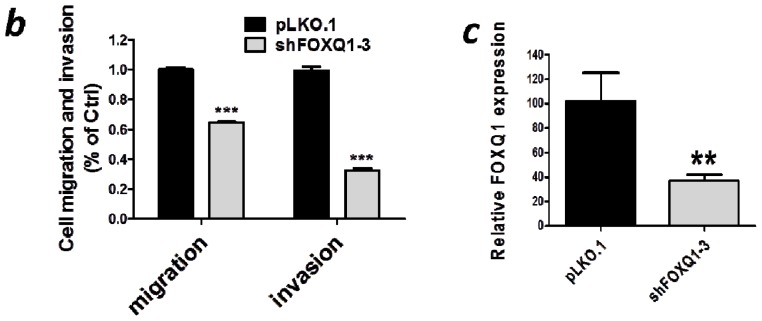
FOXQ1 knockdown inhibited ovarian cancer cell movement. SKOV3 cells were infected with control shRNA (p LKO.1) or FOXQ1 shRNA (shFOXQ1-3), and the cells were seeded into uncoated (to measure migration) and Matrigel-coated (to measure invasion) transwell inserts. Non-invading cells were wiped from the upper side of the filter, and the nuclei of invading cells were stained with crystal violet. (**a**) Representative photos of the migration and invasion results of FOXQ1 shRNA- and control shRNA-treated SKOV3 cells. (**b**) The percentage of migrated and invaded cells in FOXQ1 shRNA-treated group as compared to the control group. (**c**) The FOXQ1 knockdown efficiency in this experiment was measured by qRT-PCR. **p < 0.01, ***p < 0.001.

**Figure 5:**
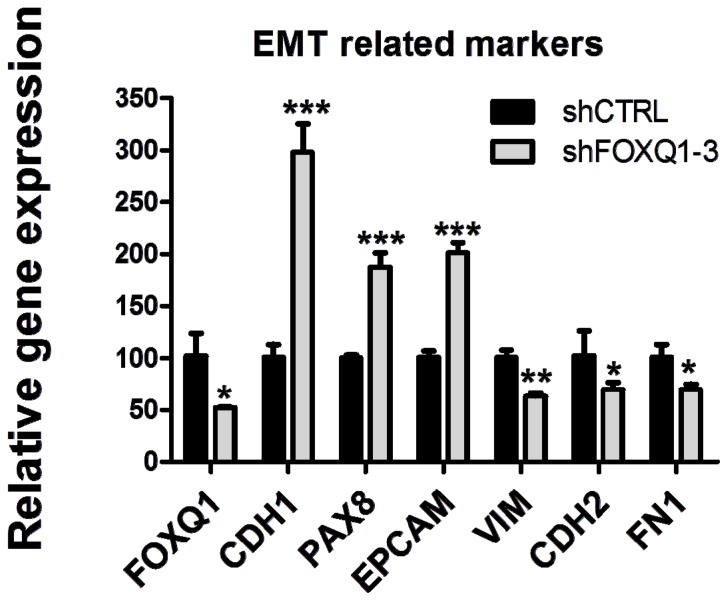
Expression levels of epithelial-mesenchymal transition (EMT) marker genes after FOXQ1 knockdown. qRT-PCR analysis indicated an increase in the mRNA levels of the epithelial markers (*CDH1*, *PAX8*, and *EPCAM*) and a decrease in the mRNA levels of the mesenchymal markers (*VIM*, *CDH2*, and *FN1*) in FOXQ1 shRNA-treated SKOV3 cells compared with control shRNA-treated cells. Relative mRNA levels were determined by using *GAPDH* as an internal control gene for data normalization. All results are presented as mean ± standard deviation. *p < 0.05, **p < 0.01, ***p < 0.001.

Given that FOXQ1 is a transcriptional factor that plays an important role in mediating EMT, which is thought to increase cellular motility and invasion, we compared both motility/invasion and EMT phenotypes observed in FOXQ1 shRNA- and control shRNA-treated SKOV3 cells. Based on a transwell assay with or without prior Matrigel coating, we observed a significant decrease in cell migration (100% *vs.* 64%, *p* < 0.001) and invasion (100% *vs.* 32%, *p* < 0.001) in FOXQ1 shRNA-treated SKOV3 cells compared with control cells (Figure 4). This reduction in cell motility and invasion was unlikely due to the growth-inhibition effect of FOXQ1, considering that the entire transwell experiment was completed within 6 h after RNAi treatment. During this short time period, no appreciable anti-proliferative effects of FOXQ1 shRNA treatment were observed. Next, we compared the expression of the following genes in FOXQ1 knockdown and control SKOV3 cells: *CHD1*, *PAX8*, *EPCAM*, *VIM*, *CHD2*, and *FN1*, which have been known to be associated with EMT. As shown in Figure 5, FOXQ1 knockdown upregulated the epithelial cell markers *CHD1*, *PAX8*, and *EPCAM* and downregulated the mesenchymal markers *VIM*, *CHD2*, and *FN1* (*p* < 0.05).

The demonstration that FOXQ1 is required for the motility and invasion of ovarian cancer cells may indicate its role in the widespread dissemination of tumor cells in the pelvic and peritoneal cavities, a well-known phenomenon in ovarian cancer. This is particularly important in high-grade serous carcinoma, which is almost always associated with numerous intraperitoneal tumor implants rather than a localized tumor at the time of diagnosis. It is plausible that expression of FOXQ1 empowers tumor cells to invade into the soft tissue underneath the mesothelial layer, facilitate further tumor growth, and ultimately lead to the establishment of tumor implants which result in incurable disease in the patients. It is important to determine whether the enhanced cellular motility and invasion is a result of epithelial mesenchymal transition (EMT) mediated by FOXQ1 overexpression. To this end, we analyzed SKOV3 cells to look for altered expression of several markers associated with EMT. We observed that FOXQ1 knockdown upregulated the epithelial cell markers *CHD1*, *PAX8*, and *EPCAM* and downregulated the mesenchymal markers *VIM*, *CHD2*, and *FN1*, a result supporting that FOXQ1 is essential for the EMT phenotype [15]. Future studies are needed to demonstrate EMT in ovarian cancer tissues and to determine the role of FOXQ1 in propelling other aggressive behaviors of cancer cells such as stem cell-like properties and acquisition of resistance to chemotherapy.

## 3. Experimental Section

### 3.1. Cell Line and Culture Conditions

The human ovarian cancer cell line SKOV3 used in this study was purchased from American Tissue Type Collection (ATCC; Rockville, MD, USA). The cells were cultured in RPMI-1640 medium supplemented with 5% (*v*/*v*) fetal bovine serum, 100 U/mL penicillin, and 100 g/mL streptomycin (Invitrogen, Grand Island, NY, USA). Cells were incubated at 37 °C with 5% CO_2_.

### 3.2. Quantitative Real-Time PCR

First-strand cDNA was synthesized using the iScript cDNA synthesis kit (Bio-Rad, Foster City, CA, USA). Relative transcript expression levels were measured using the CFX96 Real-Time PCR Detection System (Bio-Rad) and quantified by the fluorescence intensity of SYBR Green I (Invitrogen). The primers sequence for *FOXQ1* were 5′CTCAACGACTGCTTCGTCAA3′ and 5′GTGTACTCGCTGTTGGGGTT3′, E-cadherin (*CDH1*): 5′GTCCTGGGCAGACTGAATTT3′, and 5′GACCAAGAAATGGATCTGTGG3′; vimentin (*VIM*): 5′CGAGGAGAGCAGGATTTCTC3′, and 5′GGTATCAACCAGAGGGAGTGA3′; epithelial cell adhesion molecule (*EPCAM*): 5′AATGTGTGTGCGTGGGA3′ and 5′TTCAAGATTGGTAAAGCCAGT3′; paired box 8 (*PAX8*): 5′TGAGGGCGTCTGTGACAATG3′ and 5′CGGGACTCAGGGACTTGGT3′; fibronectin1 (*FN1*): 5′AAACTTGCATCTGGAGGCAAACCC3′ and 5′AGCTCTGATCAGCATGGACCACTT3′; and *N*-cadherin (*CDH2*): 5′TGTTTGACTATGAAGGCAGTGG3′ and 5′TCAGTCATCACCTCCACCAT3′. Averages of the threshold cycle number (Ct) were obtained from duplicate measurements. The relative gene expression level was calculated using the difference in Ct between the gene of interest and the control gene. Glyceraldehyde-3-phosphate dehydrogenase (*GAPDH*) was used as an internal control gene for data normalization.

### 3.3. Western Blots

Protein lysates were resuspended in Laemmli sample buffer containing 5% β-mercaptoethanol. The samples were analyzed by 4%–15% SDS-PAGE (Mini-Protean TGX Gels, Bio-Rad) and transferred onto a 0.2-μm polyvinylidene fluoride membrane (Trans-Blot® Turbo™ Midi PVDF Transfer Packs, Bio-Rad) using a semi-dry transfer apparatus (Bio-Rad). The membrane was then blocked with 5% non-fat dry milk and incubated with the appropriate antibodies. The immunoreactive proteins were detected by enhanced chemiluminescence (ECL; Thermo Scientific, Rockford, IL, USA). To ensure equal loading of proteins from each sample, we used a rabbit polyclonal anti-GAPDH antibody (G9545; Sigma, St. Louis, MO, USA) as an internal control. The other antibodies used in this study were: anti-FOXQ1 (AV39755; Sigma), anti-rabbit horseradish peroxidase (HRP, 711-035-152; Jackson Laboratories, Bar Harbor, ME, USA), anti-mouse HRP (715-035-150; Jackson Laboratories, Bar Harbor, ME, USA), anti-cyclin D1 (2926; Cell Signaling Technology, Danvers, MA, USA), anti-CDK4 (2906; Cell Signaling Technology, Danvers, MA, USA), anti-cyclin E (ab7959; Abcam, Cambridge, MA, USA), anti-p21 (2946; Cell Signaling Technology, Danvers, MA, USA), and anti- p27(2552; Cell Signaling Technology, Danvers, MA, USA).

### 3.4. FOXQ1 Gene Knockdown

Small hairpin RNAs (shRNAs) targeting FOXQ1 and the control plasmid (pLKO.1-puro vector only) were obtained from Open Biosystems. Four targeting sites were chosen for FOXQ1. Only shFOXQ1-3 (TRCN0000017925, CCAGCTCCTTCGCCATCGACA) showed more than 50% knockdown efficiency. We then purchased FOXQ1 shRNA lentiviral particles (sc-60660-v; Santa Cruz Biotechnology, Santa Cruz, CA, USA), which contain a pool of three target-specific 19–25 nt shRNAs (designated as shFOXQ1-pool). The corresponding control shRNA lentiviral particles shCTRL (sc-108080) were also purchased from Santa Cruz. To knock down FOXQ1, the cells were cultured until they reached 80% confluency and were then infected with shFOXQ1 lentiviral particles and with the accompanying negative control at the indicated times.

### 3.5. Cellular Proliferation Assay

SKOV3 cells were seeded at a density of 2000 cells/well in 96-well plates, with 5 replicate wells for each condition. Cells were lysed with 0.2% SDS after indicated days, and the cell number was counted using SYBR Green I (S7567, Invitrogen), which quantifies cellular DNA content using a fluorescence microplate reader (FLUOstar; BMG LABTECH, Durham, NC, USA). The increase in relative cell number over time was plotted against days after plating in order to track proliferation characteristics of the FOXQ1 knockdown cells.

### 3.6. Colony Formation Assay

Anchorage-dependent growth of SKOV3 cells was investigated by the monolayer colony formation assay. After infecting with the indicated shRNA lenti-viruses for 48 h, a total of 5000 cells were seeded in 6-well plates in triplicate and cultured for 14 days in complete RPMI medium containing 5% FBS. Surviving colonies were stained with crystal violet after formaldehyde fixation.

### 3.7. Flow Cytometry Assay

For analysis of cell cycle and DNA synthesis, the percentages of cells at different cell cycle stages were determined using a BD LSR II Flow Cytometer (BD Biosciences, San Jose, CA, USA). The cells were labeled with 5-ethynyl-2′-deoxyuridine (EdU) by using the Click-iT EdU kit (Invitrogen) according to manufacturer’s instructions. The cells were then labeled with Alexa Fluor 647 azide (Invitrogen), and DNA was labeled with CellCycle 405-blue dye (Invitrogen) for 30 min. The cell populations at different stages were analyzed by the CellQuest software. Three independent experiments were performed for each group.

### 3.8. Caspase Assay

Cells were seeded in 96-well plates at a density of 5000 cells/well in triplicate and treated for 36 h or 48 h with shRNAs. Caspase activity was detected using the Caspase-Glo 3/7 Assay (G8091, Promega) as per the manufacturer’s instructions. Briefly, 100 μL of the Caspase-Glo 3/7 substrate with buffer was added into each well and mixed gently. After incubation at room temperature for 30 min, the luminescence intensity of each sample was measured using the FLUOstar microplate reader (BMG LABTECH, Durham, NC, USA) and was presented as a fold-increase relative to the respective controls.

### 3.9. Migration and Invasion Assay

Transwell assay was performed to detect the migration and invasion of SKOV3 cells. FOXQ1 knockdown or control SKOV3 cells were seeded into the top chambers of 24-well plates (pore size: 8 mm; BD Biosciences, Mississauga, ON, Canada) in 250-μL serum-free medium. Uncoated inserts (354578, BD Biosciences, Mississauga, ON, Canada) were used for migration assays, whereas inserts precoated with Matrigel (354480, BD Biosciences, Mississauga, ON, Canada) were used for invasion assays. Medium with 10% FBS (750 μL) was added to the lower chamber and served as the chemoattractant. After 6 h incubation at 37 °C in 5% CO_2_, non-migrating/invading cells were removed with cotton buds from the upper side of the membrane. Cells on the underside of the membrane were fixed with 4% formaldehyde and stained with 0.1% crystal violet. The cells were then photographed at 200× magnification under phase contrast microscopy. The cells in five individual fields of each insert were counted with two wells per treatment.

### 3.10. Statistical Analysis

Statistical analysis was performed using the Prism GraphPad Prism software (GraphPad Software, San Diego, CA, USA). Data were presented as means ± standard deviations. Two-tailed *t*-tests were used to determine significance between groups. Results were considered as having a statistically significant difference when *p* < 0.05. The results were represented according to the standard Michelin guide scale (*** *p* < 0.001, ** *p* < 0.01, and * *p* < 0.05).

## 4. Conclusions

In summary, FOXQ1 is overexpressed in both tissues and cell lines of epithelial ovarian cancers compared to their normal epithelial counterparts. This is the first report of FOXQ1 upregulation in ovarian carcinomas, and more importantly, this report demonstrated the expression of FOXQ1 is essential to maintain cell cycle progression and promote cellular motility and invasion of ovarian cancer cells. The data as demonstrated in this study warrants further investigation of FOXQ1 target-based therapies for advanced stage ovarian carcinomas.
